# Size-Tailored Physicochemical Properties of Monodisperse Polystyrene Nanoparticles and the Nanocomposites Made Thereof

**DOI:** 10.1038/s41598-020-62095-8

**Published:** 2020-03-23

**Authors:** Shahin Homaeigohar, Rakibul Kabir, Mady Elbahri

**Affiliations:** 1 Nanochemistry and Nanoengineering, Department of Chemistry and Materials Science, School of Chemical Engineering, Aalto University, Kemistintie 1, 00076 Aalto, Finland; 2 Present Address: Econic Technology Ltd, Alderley Park, Macclesfield, SK10 4TG UK

**Keywords:** Materials science, Nanoscale materials, Nanoparticles

## Abstract

The latex monodisperse polystyrene (PS) colloids are important for different advanced applications (e.g. in coating, biotechnology etc.). However, the size dependency of their structural properties that impacts the characteristics of the nanocomposites composed thereof is largely unknown. Here, monodisperse PS nanoparticles (MPNPs) are synthesized via emulsion polymerization in five sizes (50, 150, 300, 350, and 450 nm). The size of the PS MPNPs is tailored by controlling the reaction time, temperature, and amount of surfactant and initiator. The correlation between the particle size and structural properties of the PS MPNPs is established by different thermomechanical and optical characterizations. The smaller particles (50 and 150 nm) show a lower glass transition (*T*_g_) and thermal decomposition temperature and a lower Raman peak intensity. Yet, they trigger a higher IR absorption, thanks to a larger surface area. When incorporated in a polyvinyl alcohol (PVA) matrix, the smaller particles impart the resulting nanocomposite a higher tensile strength, and elastic and storage moduli. Whereas, they decline the elongation and loss factor. The very few examples of the MPNPs incorporated polymeric nanocomposites have been unstudied from this perspective. Thus, these tangible knowledge can profit scalable production of this kind of nanocomposite materials for different applications in a cost/energy efficient manner.

## Introduction

The latex dispersions, i.e. the waterborne polymer colloids, show well-defined features that enable their commercial utility in various application areas such as cosmetics, drug delivery, pharmaceuticals, adhesives, coatings, inks, paints, etc.^1–3^. In such applications, the zero dimensional (0D) nanoparticles that are mainly uniform in terms of size and morphology, act as the “building blocks” of a structure with larger dimensionality. The polymer colloids, particularly in the monodisperse size regimes, hereafter called MPNPs, allow for tailoring the meso- and even macroscale arrangement of the system made thereof based on ordered structures of the nanoparticles^1^.

The MPNPs can be synthesized through various polymerization methods such as micro-emulsion, mini-emulsion, surfactant-free emulsion, and interfacial polymerization^4–6^. The emulsion polymerization is in fact the conventional synthesis technique for a variety of specialty polymers. In this technique, water that is employed as the dispersion medium is quite ecofriendly and enables efficient heat dissipation during the synthesis. Other than water, a monomer that is hardly soluble in water, a water-soluble initiator, and a surfactant are also involved in the polymerization. The initiation step takes place as soon as the monomer and initiator molecule (or ion or a free radical) meet in the continuous phase wherein the monomer is as a solute. Eventually, solid nanoparticles form via phase separation before or after termination of the polymerization reaction^7^.

Depending on the targeted application, the properties of the MPNPs need to be optimized and customized. In this regard, size plays a pivotal role and can be tuned by controlling the involved parameters in the emulsification process. For the MPNPs, the particle size is of utmost importance because it notably affects the key properties of the system composed thereof including viscosity, surface area, and packing density^7^. On the other hand, size of the nanoparticles dictates the nanoparticle’s own properties such as thermomechanical ones.

Despite a large number of studies concerning the synthesis of monodisperse polymeric nanoparticles, there have been few and almost non-existent researches showcasing the relationship between size and structural properties of the MPNPs. Here, we synthesize the polystyrene (PS) MPNPs at different sizes and for the first time, to the best of our knowledge, monitor their thermal, optical, and mechanical properties versus size. An association between the MPNPs size and their thermomechanical and optical properties can later profit the relevant applications. For instance, development of coatings based on the polymer nanoparticles that are free of volatile organic compound (zero-VOC coating) and thus ecofriendly could be achieved through optimizing glass transition temperature (*T*_*g*_) versus the particle size^8^. VOCs are utilized in order to reduce the *T*_*g*_ of the particle, thereby easing the manufacture of the films that are resistant against cracking. In case, *T*_g_ is declined simply by changing the particle size, there would be no need to use of VOC. Another example is the fabrication of the nanocomposites whose mechanical properties can be tailored by inclusion of polymeric nanoparticles in different morphologies, sizes, and filling factors.

In this study, not only for the first time, the correlation between the size of the PS MPNPs and their thermomechanical and optical properties is investigated, but also via a simpler, and less energy consuming approach compared to the melt compounding, an elastomeric (polyvinyl alcohol; PVA based) nanocomposite is made. This synthesis enables uniform dispersion and orientation of the PS MPNPs within the polymer matrix, readily by mixing the particles suspension in the polymer matrix aqueous solution. The fabrication method of the mentioned nanocomposite is not novel *per se* but allows for associating the nanoparticle size and mechanical properties of the as-synthesized material. The knowledge extracted from this investigation can be beneficial for large scale production of this kind of nanocomposites for different applications in a cost/energy efficient manner.

## Results and Discussions

### Morphology of the synthesized nanoparticles

**Figure 1:**
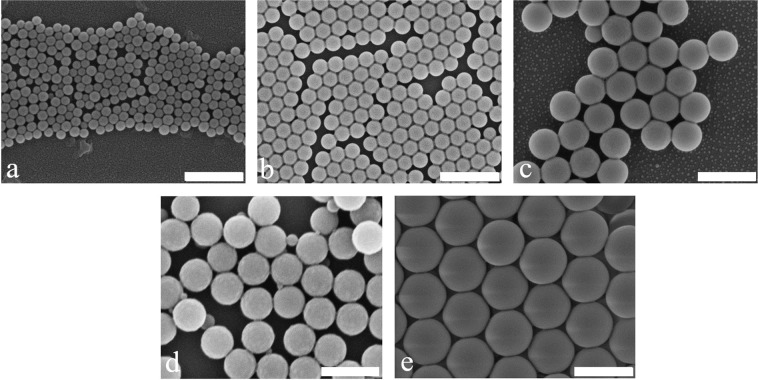
SEM images show the PS MPNPs at different particle sizes of: (**a**) PS1 (50 ± 6 nm), (**b**) PS2 (150 ± 12 nm), (**c**) PS3 (300 ± 8 nm), (**d**) PS4 (350 ± 6 nm), and **(e**) PS5 (450 ± 10 nm) (The scale bar represents 500 nm). The particle sizes were measured by Delsa Nano C, DLS, and SEM images (by using software Digital Micrograph Demo 6.3.5). The obtained results were averaged.

The morphology of the PS MPNPs in different sizes is shown in Fig. 1a–e. While the majority of the MPNPs including PS1, PS2, PS3, and PS5 are totally monodisperse, PS4 (Fig. 1d) is an exception and contains a negligible amount of smaller nanoparticles.

### Size dependent thermal properties of the nanoparticles

**Figure 2:**
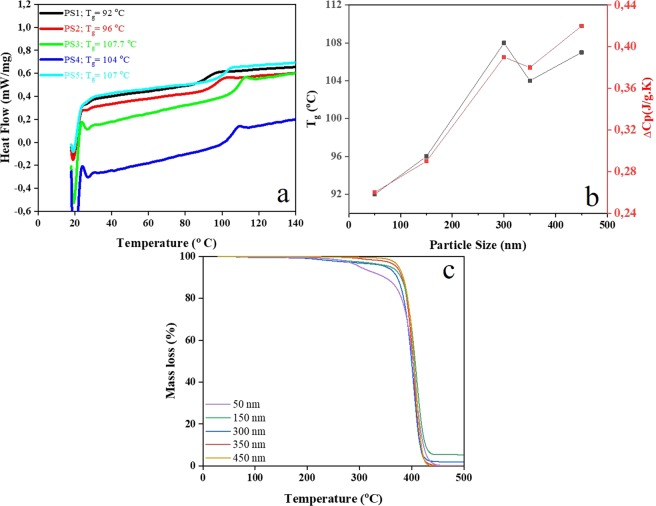
Thermal properties of the PS nanoparticles. (**a**) DSC spectra imply the rise of T_g_ with increase of the particle size; (**b**) The T_g_ and C_p_ values extracted from the DSC spectra explicitly demonstrate the direct correlation between the nanoparticle size and thermal transition temperature and the relevant released heat; (**c**) TGA spectra imply the lower thermal degradation temperature of the PS nanoparticles with smaller sizes.

Figure 2a shows the DSC curves for the PS MPNPs with different sizes. Based on these curves, the glass transition temperatures (T_g_s) corresponding to each size was measured using the midpoint method by intersecting the DSC trace. The determined values are shown in Fig. 2b and tabulated in Table 1. Additionally, the increments of the specific heat capacity taking place at T_g_ are presented in Fig. 2b. It is apparently seen that T_g_ rises proportionally with the particle size from 92 to 108 °C.

**Table 1:** Glass transition temperature of the PS nanoparticles as alone and as incorporated within a PVA matrix, measured by DSC and DMA method, respectively.

Sample	Particle size (nm)	T_g_ (PS MPNP- DSC) (°C)	T_g_ (PS MPNP/PVA -DMA) (°C)	
			γ transition	α transition
PS1	50	92	42	101
PS2	150	96	36.6	103
PS3	300	107.7	34.5	105
PS4	350	104	44.2	110
PS5	450	107	44.4	103

The γ and α transition temperatures for the nanocomposite system are also included in the table.

The higher T_g_ for the larger nanoparticles (PS3, PS4, and PS5) could be attributed to their less exposed surface area and thus lack of sensitivity to heating compared to the smaller ones (PS1 and PS2). Accordingly, heat absorption by the larger nanoparticles takes place more slowly and the glassy to rubbery transition proceeds with much higher energy consumption. While the PS1 MPNPs require only 266 J to undergo the transition, the PS5 ones cost 414 J. Such a finding i.e. the correlation of T_g_ and specific heat capacity to the nanoparticle size has been previously declared by other researchers, as well^9,10^.

The relationship between the ΔC_p_(r) (i.e. the heat capacity difference between the glassy and rubbery state) and T_g_(r) with the nanoparticle size (r) can be explained through the following Eqs. (1 and 2)^10^:

1 $$\frac{\Delta {C}_{p}(r)}{\Delta {C}_{p}(\infty )}=1-\frac{1}{\frac{r}{{r}_{0}}-1}$$

2 $${T}_{g}(r)\times {T}_{g}(\infty )=exp-2[\Delta {C}_{p}(\infty )-\Delta {C}_{p}(r)]3R.$$

where $\Delta {C}_{p}(\infty )$ is the heat capacity difference between the bulk glass and bulk liquid at T_g_(*∞*). r_0_ is the critical radius whereat all atoms of the nanoparticle are located on its surface. R denotes the ideal gas constant. The above equations clearly verify that for the MPNPs with a smaller size (r), given a constant r_0_, R, *T*_g_(*∞*) and $\Delta {C}_{p}(\infty )$, a lower ΔC_p_(r) and T_g_(r) could be expected.

TGA was also carried out to scrutinize the degradation behavior and thermal stability of the PS MPNPs when subjected to high temperatures. Figure 2c shows the TGA curves for the PS MPNPs at a heating rate of 20 K.min^−1^. As seen here, the thermal degradation of the PS MPNPs occurs mainly in the range of 260–470 °C, depending on the particle size. While the degradation of 50 nm PS MPNPs starts at 250 °C (T_onset_) (with a weight loss of 2.85%), the 450 nm PS MPNPs undergo a degradation process at 350 °C (T_onset_) (with a primary negligible weight loss of 0.3%). This discrepancy implies the higher thermal stability of the larger PS MPNPs, most likely due to their lower exposed surface area. The weight loss goes on up to 440 °C thereafter the sample weight remains fixed, implying ash formation. An identical size dependency of thermal degradation behavior for nanoparticles has been also reported by Mohamed *et al*.^11^ though for a ceramic material.

### Size dependent surface phonon modes of the nanoparticles

Raman spectroscopy is a reliable technique for exploration of the structural characteristics of nanoparticles as a function of their size. Any change in the Raman spectra with decreases in particle size can be readily tracked. In fact, the Raman analysis not only enables determination of nanoparticle size, but also provides information about the surface modes of the nanoparticle caused by its finite size. With respect to the latter, the surface modes prevail with the particle size decline and enlargement of the surface-to-volume ratio, thereby emerging new bands^12^.

**Figure 3:**
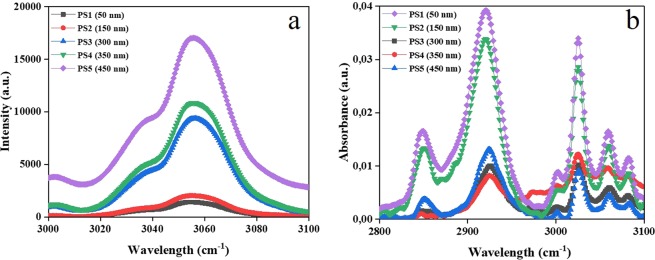
(**a**) Raman and (**b**) ATR-FTIR spectra for the PS MPNPs in different sizes.

Figure 3a shows the Raman spectra for the PS MPNPs within the range of 3000–3100 cm^−1^ representing the aromatic CH stretching (at 3055 cm^−1^)^13^. As seen here, the peak intensity declines and the Raman bands are broadened with the nanoparticles’ shrinkage. Such a behavior has been previously reported by Choi *et al*.^12^. As they state, for smaller TiO_2_ nanoparticles, the Raman bands shift to higher wavenumbers and their intensities relatively decrease. When the particle size lies in the nanometer scale, the vibrational properties undergo two important changes. First, a volume contraction takes place within the nanoparticle by the size-induced radial pressure. This structural change raises the force constants as a result of the lower interatomic spacing. Second, the nearest neighboring shells are notably affected by the particle’s shrinkage. This effect gives rise to increment of the mean square relative displacement (MSRD), thereby declining the vibrational amplitudes of the nearest neighbor bonds^14^, as similarly recorded in our study.

### Size dependent optical properties of the nanoparticles

FTIR is another vibrational spectroscopy technique that provides useful information regarding the chemical structure of a polymeric system. Any particle size dependent change in the FTIR spectra could be attributed to alteration in vibrational modes and atom-atom bonding caused by size induced re-arrangements.

Figure 3b shows the FTIR absorbance spectra for the PS MPNPs in the range of 2800–3100 cm^−1^. In this selected zone, the peak appearing at 2940 cm^−1^ is characteristic of the out-of-plane bending vibration of C-H in the benzene ring^15^. Apparently, the nanoparticles with the sizes of 50 and 150 nm, i.e. PS1 and PS2 show a much larger IR absorption compared to the rest of the particle sizes. Such a size-dependent optical property of the PS MPNPs whose size is around a few hundred nanometers, is totally different from the “quantum confinement effect”, typically seen in the semiconductor ultra-small particles (smaller than 10 nm in diameter)^16^. On the other hand, the Mie theory, traditionally used for metal nanoparticles, does not apply here. In fact, the Mie scattering justifies the shift in the absorption peak’s position rather than its broadening or intensification. The small nanoparticles’ intensive IR absorption could be attributed to the extraordinary surface area of these nanoparticles involving a higher density of the surface constituting atoms or altering the lattice state^17^. The enhancement of the surface area provokes lattice softening and thereby reduces the intermolecular Coulombic interaction energies and expands the band gaps. In analogue to our results, the impact of particle size on IR absorption of other organic nanoparticles including 1-phenyl-3-((dimethylamino)styryl)-5-((dimethylamino)phenyl)-2-pyrazoline (PDDP)^16^ has also been reported.

### Size dependent thermomechanical properties of the nanoparticulate composites

PVA is an eco-friendly, inexpensive, non-toxic, and biocompatible polymer and has extensively attracted research attention for development of coating, nanofiber, and bulk materials^18^. Particularly, the PVA nanocomposites have been appealing for a wide range of applications including biomedicine^19^, gas separation^20^, resistive switching^21^, opto-electronics^22^ among others. Despite the remarkable potential of the PVA nanocomposites, it is mechanically weak and needs to be reinforced^18^. In this regard, controlled construction of the material by engineering of the nanofillers’ characteristics e.g. to induce the desired thermomechanical properties is challenging. Here, we incorporate the PS MPNPs with different sizes into PVA to optimize its mechanical properties while correlating the nanoparticle size and the mechanical properties of the PVA nanocomposite. As mentioned earlier, the obtained results can be favorable for the scalable production of this kind of nanocomposite materials for a variety of applications in a cost/energy efficient manner.

**Figure 4:**
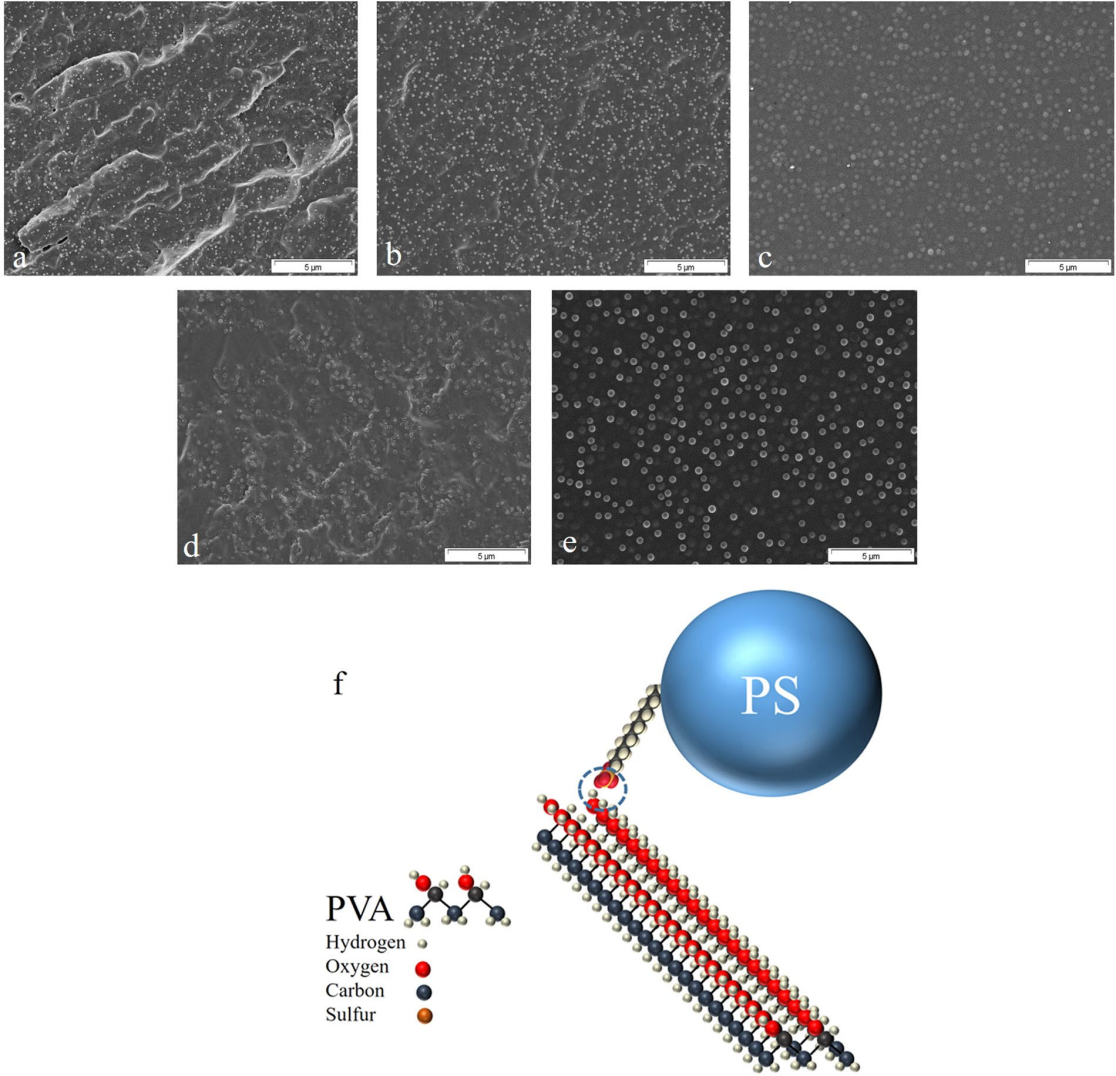
SEM micrographs demonstrate the distribution mode of the PS MPNPs (**a**: 50 nm, **b**: 150 nm, **c**: 300 nm, **d**: 350 nm, and **e**: 450 nm) across the PVA matrix; (**f**) Schematic illustration of the likely interaction (H-bonding, marked by a circle) between the surfactant (SDS) and PVA molecules (drawn ideally consecutively in cis-format).

Figure 4a–e shows the morphology and distribution mode of the PS MPNPs within the PVA matrix. As seen in the SEM images, the nanoparticles are uniformly distributed across the PVA matrix. There are two main factors governing the uniform distribution of the PS MPNPs. From one hand, the compounding method and water evaporation lead to regular arrangement of the particles. On the other hand, it is assumed that the remaining surfactant on the surface of the nanoparticles can optimally interact with the polar functional groups of PVA (e.g. via hydrogen bonding), Fig. 4f. Cooperatively, the two factors give rise to a homogenous dispersion of the nanoparticles within the matrix. This characteristic is quite promising for isotropicity of the structural properties of the resulting nanocomposite. It is worthy to note that in many technical applications, nanoparticles tend to agglomerate, thereby forming clusters whose size exceeds nano-regime. Accordingly, by the presence of such large clusters, violating the nanoparticles behavior, the interfacial area, i.e. the zone wherein the interaction between the filler and the polymer takes place, declines notably^23^. As a result, the volume fraction of the interphase shrinks and its impact on the mechanical properties of the nanocomposite is neutralized. Additionally, induced by the agglomeration, the nanoparticles accumulate in specific regions and the other areas of the polymer remain unoccupied and their properties unaffected^24–28^. Therefore, homogenization of the nanoparticles distribution in a polymer matrix is of great importance.

To mechanically characterize the PS/PVA nanocomposites, tensile test and DMA were considered to image the mechanical performance of the materials under static and dynamic loading modes. The tests were aimed at correlating the mechanical properties of the nanocomposite to the particle size of the inclusion and also probing how stable and robust the interface of the nanoparticles and the matrix is.

**Figure 5:**
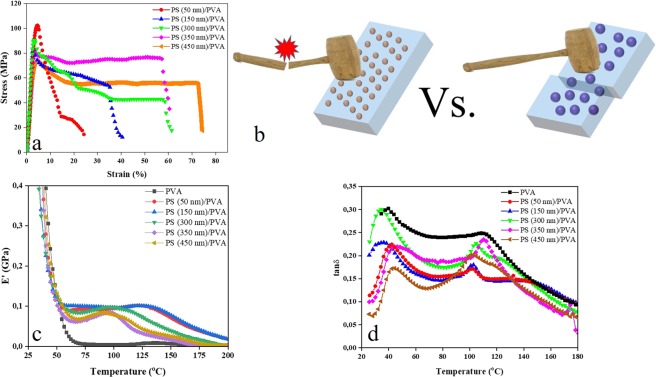
Mechanical properties of the PS MPNP/PVA nanocomposite. (**a**) tensile test results; (**b**) The schematic implies higher mechanical resistance of the nanocomposite comprising the smaller MPNPs versus the one with larger MPNPs. (**c**) storage modulus, and (**d**) loss factor of the nanocomposites.

Table 2 and Fig. 5a show the tensile properties of the samples including Young’s modulus, E, tensile strength (σ_M_), and elongation (ε_M_). According to Table 2, the nanocomposites are notably superior to the neat PVA, in terms of elastic modulus and tensile strength. However, they exhibit a significantly lower elongation at break. This optimized mechanical performance is mainly attributed to the strengthening effect of the MPNPs included that challenge chain mobility of the polymer matrix^29,30^. The nanoparticles act as physical barriers and the likely hydrogen bonding between them and the matrix, mediated by the surfactant exacerbates the deformability of the nanocomposites. Among the nanocomposites, as seen in Fig. 5a, both the E modulus and tensile strength rise by decrease of the PS MPNPs’ size. As seen in Table 2, tensile strength and elastic modulus of the sample with 50 nm MPNPs are 101 and 3950 MPa, respectively. Whereas, such quantities for the sample with 450 nm MPNPs are 72 and 3360 MPa, respectively. In contrast to elastic modulus and tensile strength, elongation declines for the samples with the PS MPNPs smaller than 300 nm. The fracture elongation ranges from 23% to 75% for the samples containing small (50 nm) and large (450 nm) particles, respectively. Such mechanical performances could be attributed to the discrepancy in the number and the nature of the MPNPs distributed in the PVA matrix. The smaller particles are stiffer and outnumbers the larger ones. Accordingly, a more extensive area of the matrix is in direct contact with the small MPNPs rather than the larger ones, resulting in a higher elastic modulus and tensile strength. However, given the relatively poor bonding (via secondary intermolecular forces) of the PS MPNPs and PVA, the surrounding area of each particle is considered as a void, in fact, and a cracking initiation point. Accordingly, the smaller, stiffer particles endow the matrix with a higher robustness and stiffness, because they restrict the matrix’ mobility and deformation by applying a mechanical restraint. As Fu *et al*.^31^ state the polymer chains’ mobility is exacerbated in the proximity of the nanoparticles thanks to a notable attraction tendency between the chain’s segments and the repulsive potential imposed to the polymer from the adjacent nanoparticles. Despite improvement of stiffness, the small nanoparticles form a higher density of voids, engendering lower elongation. While the tensile forces is applied, crazes start to emerge as soon as the stress exceeds a critical value. In general, in the case of such nanocomposite systems, where efficient load transfer between the filler and the matrix is impeded, de-wetting across the phase boundary shapes a tiny cap-like cavity over the nanoparticle. Such a cavity provokes additional stress concentration around its sharp edge, engendering initiation of craze with a lower stress expense as compared to the nanocomposites with higher interfacial strength with no de-wetting^31^. Figure 5b schematically demonstrates the superior mechanical performance of the nanocomposites containing smaller MPNPs. In this regard, there have been several models that confirm the positive impact of particle size on tensile strength in the multicomponent polymeric systems as ours. For instance, it has been shown that the strength of silica filled epoxy increases with shrinkage of the filler size as (Eq. 3)^32^:

3 $${\sigma }_{c}={\sigma }_{m}+{k}_{p}({V}_{p}){d}_{p}^{-1/2}$$

where *σ*_c_ and *σ*_m_ denote the strength of the composite and matrix, respectively, *V*_p_ represents the particle volume fraction and *k*_p_(*V*_p_) is a constant that depends on the volume fraction, *d*_*p*_ also denotes the particle size (diameter).

**Table 2:** Tensile properties of the PS MPNP/PVA samples including E-modulus, *E*, elongation, ε_M_, and the tensile strength, σ_M_ (The values related to PVA (same grade as ours) were extracted from^39^).

Sample	E Modulus (MPa)	σ_M_ (MPa)	**ε**_**M**_**(%)**
PVA	210 ± 15	25.3 ± 4.3	250 ± 10
PS(50 nm)/PVA	3950 ± 20	101.1 ± 5	23 ± 4
PS(150 nm)/PVA	3600 ± 26	84 ± 3	40 ± 5
PS(300 nm)/PVA	3450 ± 8	90.3 ± 2	61 ± 3
PS(350 nm)/PVA	3100 ± 20	80.2 ± 3	60 ± 3
PS(450 nm)/PVA	3360 ± 15	72.7 ± 5	75 ± 2

Such a correlation is also defined in a different way for a similar silica filled epoxy composite as (Eq. 4)^33^:

4 $${\sigma }_{c}={\sigma }_{m}+S/{D}_{s}$$

where S is a constant and Ds i.e. the interparticle distance is expressed as (Eq. 5):

5 $${D}_{s}=2{d}_{p}(1-{V}_{p})/3{V}_{p}$$

Apparently, both the models imply that a smaller nanofiller size confers the related composite with a larger tensile strength, as we observed in our nanocomposite.

**Figure 6:**
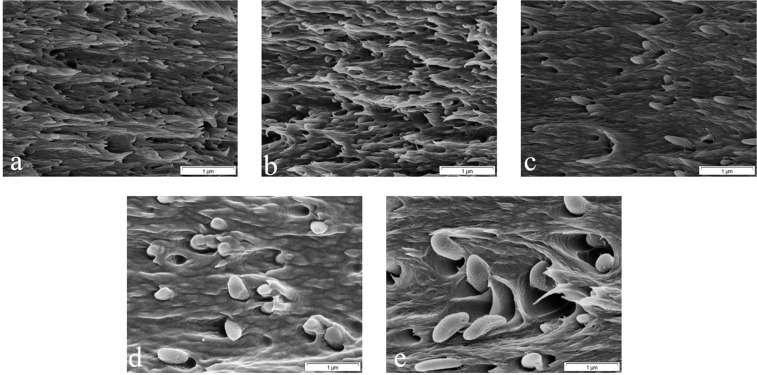
SEM image of the cross-sectional fracture surfaces of the PS MPNP/PVA nanocomposite broken in the tensile test (**a–e** represent the nanocomposites containing 50, 150, 300, 350, and 450 nm PS MPNPs, respectively).

SEM images of the fracture surface of the samples, Fig. 6a,b, imply that a more brittle fracture takes place for the samples with smaller PS MPNPs. With respect to the larger MPNPs, the number of voids is notably less and they can partly get involved in the stretching process alongside the polymer matrix. Figure 6c–e clearly indicates elongation of the particles (as much as 1 µm) alongside the polymer. Thus, cooperatively, the PS/PVA nanocomposites encapsulating larger particles show higher elongation of up to 75%. In contrast, when using small particle sizes, de-bonding takes place and voids form that hamper stress transfer at the particle/polymer interface^34,35^. Similarly, a same effect of particle size and particularly the small sized particles on viscoelastic properties and rheology of an acrylic polymer has been previously reported^36^.

The effect of the particle size on the dynamic mechanical properties of the PVA nanocomposites can be monitored by DMTA while they are heated, and through quantities such as loss factor (*tanδ*) and storage modulus (*E*’).

Figure 5c shows the temperature dependent variation for storage modulus (*E*’) of the PS MPNP/PVA nanocomposites. Apparently, the storage modulus for all the samples declines at 60–75 °C due to the glass transition of the PVA matrix. Compared to PVA, the nanocomposites reach minima at a slightly lower temperature, while their minima takes place at a larger modulus. This observation indicates the higher stiffness of the nanocomposites, especially the ones containing smaller MPNPs and on the other hand the lower energy required for segmental mobility, likely due to presence of voids enabling expansion of the structure. The bump emerged in the rubbery plateau for the nanocomposites is arisen from the reinforcing effect of the nanoparticles for the polymer matrix^37^, which is notably higher for the smaller nanoparticles. As stated earlier, the smaller nanoparticles provide a larger surface area thus cause a higher interaction with the matrix.

The loss factor, tan δ, i.e. the ratio of loss modulus (E”) to the storage modulus (E’), describes the relaxation processes in PVA and PS MPNP/PVA nanocomposites. Figure 5d shows the variation of tanδ of the PVA nanocomposites versus temperature. Based on these spectra, Tg can be assessed where tanδ peaks. Surprisingly, inclusion of the PS MPNPs shifts the tanδ peaks slightly to lower temperatures. The magnitude of this shift is dependent on the particle size so that smaller particles trigger a larger shift. While heating, the free volume of the chain segment and chains’ ability to move in various directions increase. Depending on the extent and range of mobility, two main thermal transitions of α and γ are defined. Typically the γ transition occurs at a lower temperature, in contrast to the α transition or glass transition leading to the rubbery state of the polymer. According to Fig. 5d and as tabulated in Table 1, for the PVA film the γ and α transitions take place at around 39.6 °C and 110.5 °C. With respect to the PVA nanocomposites, γ and α transitions are triggered at 34–45 °C and 100–110 °C, respectively, depending on the particle sizes. Having one α transition peak for the PVA nanocomposites could point out to a partial miscibility or some kind of interaction between the nanoparticles and the matrix^37^. This conclusion is further verified when considering uniform distribution of the nanoparticles across the matrix (Fig. 4a–c). While no significant contribution is seen on transitions of PVA by the nanoparticles, Fig. 5d shows that the loss factor i.e. the relaxation peak declines for the nanocomposite versus the neat PVA. Such a loss is intensified for the nanocomposites containing smaller MPNPs, due to higher stiffness (storage modulus) of this group of the samples, arisen from the extensive surface area of this sort of nanoparticles in touch with the matrix.

As deduced from Fig. 5c,d, the glass transition behavior of the PS MPNP/PVA nanocomposites is relatively unpredictable versus that monitored in DSC. This could be due to this fact that in DMTA, the variation of tan*δ* in the glass-to-rubber softening region is influenced not only by the local segmental motions in the loss modulus at a lower temperature, but also by filler reinforcement effects on both the storage and loss modulus at higher temperatures. In the literature, there are many controversial reports concerning the effect of inclusions on the glass transition of the polymer matrix. While a few couple of studies point out to enhancement of *T*_g_ by inclusion of carbon black, silica, or other particles, there are several others that show no notable effect on *T*_g_ or even loss of *T*_g_^38^. Conclusively, the extent and nature of the interfacial interactions between the polymer and inclusions might play a decisive role in such behaviors.

## Conclusion

Here, we successfully synthesized monodisperse polystyrene nanoparticles in different particle sizes through emulsion polymerization. Subsequently, the structural properties of the nanoparticles including optical and thermomechanical ones were characterized to elaborate the size-properties association. It was shown that T_g_ and the Raman peak intensity decline with lowering the particle size. In contrast, IR absorption rises when the particle size shrinks. The as-synthesized PS nanoparticles were incorporated within a PVA matrix to investigate their impact (in different sizes) on the mechanical properties of the resulting material. The tensile strength and E-modulus raised more notably for the smaller nanoparticles, while elongation was higher for the larger nanoparticles. Such discoveries were further verified by DMTA and under a dynamic load. The nanocomposites containing smaller nanoparticles showed a higher storage modulus and lower loss factor.

## Experimental Section

### Materials

The styrene monomer was purchased from Sigma-Aldrich (US), purified by passing through porous silica and eventually stored at 4 °C. Sodium dodecyl sulfate (SDS) (≥98.5% GC), sulfuric acid (ACS reagent, 95–98%), polyvinyl alcohol (Mowiol 10–90, M_w_ = 61,000), and potassium persulfate (ACS reagent, ≥99%) were purchased from Sigma-Aldrich. The ultrapure water used in the experiments was generated by an Ultra Clear UV Plus purification system (SG wasseraufbereitung und regenerier station, GmbH) with conductivity of 0.055 µS/cm. Hydrogen peroxide (30% for analysis) and ammonium hydroxide (~24% in water) were purchased from Merck KGaA and Fluka analytical, respectively.

### Characterizations

The average particle size and size distribution of the PS MPNPs were determined by a particle size analyzer (DelsaNano C particle size analyzer, Beckman Coulter, USA) operating based on the photon correlation spectroscopy (PCS) method. The particle size of the PS MPNPs was further measured through the Dynamic Light Scattering (DLS) technique (ALV-Laser Vertriebsgesellschaft. m.b.H., Langen, Germany). The measurements were performed at room temperature by using a vertically polarized 22 mW laser light with the wavelength of 632.8 nm. The morphology of the PS MPNPs was assessed by SEM (LEO Gemini 1550 VP, Zeiss) at a 10 kV accelerating voltage. Beforehand, the particles were sputter-coated with gold. The size of the PS MPNPs imaged by SEM was also quantified using the software ‘Digital Micrograph Demo 6.3.5.

To evaluate the thermal properties of the PS MPNPs, Differential Scanning Calorimetry (DSC) was performed. To do so, the equipment (Netzsch DSC Phoenix) was calibrated using indium and cyclohexane. The standard aluminum pans (50 μL) were employed to hold the samples (10 mg ± 1 mg) subjected to dynamic heating and cooling scans. The thermal treatments were carried out under the nitrogen atmosphere at the rate of 20 K/min. A second heating process was applied to assess the thermal transitions. The thermogravimetric analysis (TGA) measurements were also done using the Netzsch TG209 F1 Iris instrument. The readings were obtained under a constant argon flow (20 mL/min) at a heating rate of 5 °C/min.

To characterize the surface chemistry of the PS MPNPs, Fourier transform infrared spectroscopy (FT-IR) was conducted using the Bruker Equinox 55 machine. The Raman spectroscopy of the particles was also done using the Senterra Raman Bruker Optics instrument. In these measurements, the exciting laser’s wavelength was 532 nm.

The mechanical properties of the PS MPNP reinforced PVA nanocomposites were quantified by a uniaxial tensile tester (Zwick model Z020, with a load cell of 0.5 N). The measurements were done under a cross-head speed of 5 mm/min at ambient temperature according to ASTM D882. The dynamic mechanical analysis (DMA) was also performed at the temperature range of 20 °C to 200 °C with a heating rate of 3 K/min. The applied frequency was 10 Hz and a fixed 1 N force was exerted for every sample.

### Sample preparation

#### The PS MPNPs synthesis

The PS MPNPs were synthesized via emulsion polymerization under the desired conditions, tabulated in Table 3.

**Table 3:** Various polymerization conditions leading to different nanoparticle sizes.

Sample code	Surfactant (SDS) (g)	Initiator (KPS) (g)	Water (ml)	Temperature (°C)	Duration (h)	Particle size (nm)
PS5	—	0.03	300	70	29	450
PS4	—	0.1	300	70	20	350
PS3	—	0.3	300	70	24	300
PS2	0.3	0.1	200	90	18	150
PS1	0.1	0.05	100	90	8	50

The polymerization reactions were carried out in a 300 ml round-bottom one-necked flask entirely or partially filled with distilled water (according to Table 3). The flask was exposed to N_2_ flow for 30 minutes to deoxygenate the water. Afterwards, a water cooled reflux condenser was installed at the outlet of the flask. The flask was then immersed in an oil bath equipped with a thermometer. At this stage, the surfactant was added to the flask and the temperature was increased up to 80 °C. Upon reaching the desired temperature, the purified styrene was poured into the reaction mixture. The mixture was then deoxygenated by passing N_2_ flow (for 30 min). The polymerization was initiated by including the initiator at 80 °C. During the course of polymerization, the reaction medium was being steadily exposed to the nitrogen gas. Eventually, the latex dispersions of polystyrene with various particle sizes of 50 to 450 nm, depending on the parameters tabulated in Table 3, were resulted.

#### Preparation of Silicon wafers

A Si (100) plate was cut into 1 cm × 1 cm wafers, that were hydrophilized as following: First, the Si wafers were cleaned with the piranha solution (i.e. a mixture of sulfuric acid and hydrogen peroxide at the ratio of 3:1) at 80 °C for 1 hour, followed by rinsing copiously with deionized water and immediate drying with N_2_ gas. After cleaning, a mixture solution of deionized water, ammonium hydroxide, and hydrogen peroxide was prepared at the ratio of 5:1:1 wherein the Si wafers were immersed and boiled at 80 °C for 1 hour. Eventually, the wafers were washed thoroughly and kept in 10% sodium dodecyl sulfate solution for 24 h.

#### Deposition of the PS MPNPs onto the Si wafers

A single layer of the PS MPNPs was deposited onto Si wafers by spin coating. The as-prepared samples were used for Raman and SEM measurement. To do so, 100 µl PS suspension was spin coated on a Si wafer at 3000 rpm for 30 sec and then oven dried before further analysis. For the other tests, including DSC, TGA, and FTIR measurements, the PS MPNP containing suspensions were air dried and subsequently tested. For DMA and tensile testing, the PS MPNPs were blended with a PVA aqueous solution. For this sake, 10 mg of the PS MPNPs and 100 mg of PVA were mixed in 25 ml distilled water. The resultant solution was poured into Petri dishes and the solvent was allowed to evaporate slowly over 3–4 weeks at 40 °C to provoke homogenous distribution of the MPNPs in the PVA matrix. The as-made nanocomposite films were then dried under vacuum at 40 °C for 1 day. To exclude the residual water, the temperature was raised to 100 °C and the samples were left to be dried one more day, followed by cooling down to room temperature. The samples prepared for mechanical tests had an average dimension of 12 mm × 35 mm × 0.25 μm.

## References

[1] Oliveira AM, Guimarães KL, Cerize NNP (2015). The role of functional monomers on producing nanostructured lattices obtained by surfactant-free emulsion polymerization – A novel approach. European Polymer Journal.

[2] Wang T, Keddie JL (2009). Design and fabrication of colloidal polymer nanocomposites. Advances in Colloid and Interface Science.

[3] Lu Y, Liu X, Luo G (2017). Synthesis of polystyrene latex via emulsion polymerization with poly(vinyl alcohol) as sole stabilizer. Journal of Applied Polymer Science.

[4] Pei X (2017). Synthesis of monodisperse starch-polystyrene core-shell nanoparticles via seeded emulsion polymerization without stabilizer. Polymer.

[5] Zhao Y, Wang H, Zhu X, Möller M (2017). One-pot formation of monodisperse polymer@ SiO 2 core–shell nanoparticles via surfactant-free emulsion polymerization using an adaptive silica precursor polymer. Polymer Chemistry.

[6] Ishii H, Kuwasaki N, Nagao D, Konno M (2015). Environmentally adaptable pathway to emulsion polymerization for monodisperse polymer nanoparticle synthesis. Polymer.

[7] Rao JP, Geckeler KE (2011). Polymer nanoparticles: Preparation techniques and size-control parameters. Progress in Polymer Science.

[8] Rharbi Y (2008). Reduction of the glass transition temperature of confined polystyrene nanoparticles in nonablends. Physical Review.

[9] Jiang Q, Shi H, Li J (1999). Finite size effect on glass transition temperatures. Thin Solid Films.

[10] Zhang Z, Shi H, Jiang Q (2000). Glass transition of organic nanoparticles. Materials Letters.

[11] Mohamed M, Yusup S, Maitra S (2012). Decomposition study of calcium carbonate in cockle shell. Journal of Engineering Science and Technology.

[12] Choi HC, Jung YM, Kim SB (2005). Size effects in the Raman spectra of TiO2 nanoparticles. Vibrational Spectroscopy.

[13] Matsushita A (2000). Two-dimensional Fourier-transform Raman and near-infrared correlation spectroscopy studies of poly(methyl methacrylate) blends: 1. Immiscible blends of poly(methyl methacrylate) and atactic polystyrene. Vibrational Spectroscopy.

[14] Rockenberger J (1997). EXAFS Studies on the Size Dependence of Structural and Dynamic Properties of CdS Nanoparticles. The Journal of Physical Chemistry B.

[15] Shimpi NG, Borane M, Mishra S (2016). TiO2/polystyrene core–shell nanoparticles as fillers for LLDPE/PLA blend: development, and morphological, thermal and mechanical properties. Polymer Bulletin.

[16] Fu H-B, Yao J-N (2001). Size Effects on the Optical Properties of Organic Nanoparticles. Journal of the American Chemical Society.

[17] Seoudi R, Shabaka AA, Kamal M, Abdelrazek EM, Eisa WH (2012). Dependence of structural, vibrational spectroscopy and optical properties on the particle sizes of CdS/polyaniline core/shell nanocomposites. Journal of Molecular Structure.

[18] Valiya Parambath S, Ponnamma D, Sadasivuni KK, Thomas S, Stephen R (2017). Effect of nanostructured polyhedral oligomeric silsesquioxane on the physical properties of poly(vinyl alcohol). Journal of Applied Polymer Science.

[19] Koosha M, Mirzadeh H, Shokrgozar MA, Farokhi M (2015). Nanoclay-reinforced electrospun chitosan/PVA nanocomposite nanofibers for biomedical applications. RSC Advances.

[20] Jahan Z, Niazi MBK, Hägg M-B, Gregersen ØW (2018). Cellulose nanocrystal/PVA nanocomposite membranes for CO2/CH4 separation at high pressure. Journal of Membrane Science.

[21] Rehman, M. M. *et al*. Resistive Switching in All-Printed, Flexible and Hybrid MoS2-PVA Nanocomposite based Memristive Device Fabricated by Reverse Offset. *Scientific Reports* **6**, 36195, 10.1038/srep36195, https://www.nature.com/articles/srep36195#supplementary-information (2016). 10.1038/srep36195 PMC5095886 27811977

[22] Karthikeyan B, Pandiyarajan T, Mangalaraja RV (2016). Enhanced blue light emission in transparent ZnO:PVA nanocomposite free standing polymer films. Spectrochimica Acta Part A: Molecular and Biomolecular Spectroscopy.

[23] Homaeigohar S, Tsai T-Y, Young T-H, Yang HJ, Ji Y-R (2019). An electroactive alginate hydrogel nanocomposite reinforced by functionalized graphite nanofilaments for neural tissue engineering. Carbohydrate Polymers.

[24] Rempe A-S (2019). Quantification of the three-dimensional nanoparticle distribution in polymer nanocomposites. IEEE Transactions on Dielectrics and Electrical Insulation.

[25] Homaeigohar S, Botcha NK, Zarie ES, Elbahri M (2019). Ups and Downs of Water Photodecolorization by Nanocomposite Polymer Nanofibers. Nanomaterials.

[26] Homaeigohar SS, Elbahri M (2012). Novel compaction resistant and ductile nanocomposite nanofibrous microfiltration membranes. Journal of colloid and interface science.

[27] Homaeigohar SS, Mahdavi H, Elbahri M (2012). Extraordinarily water permeable sol gel formed nanocomposite nanofibrous membranes. Journal of Colloid and Interface Science.

[28] Elbahri M (2012). Smart Metal-Polymer Bionanocomposites as Omnidirectional Plasmonic Black Absorbers Formed by Nanofluid Filtration. Advanced Functional Materials.

[29] Sadi AY, Homaeigohar SS, Khavandi AR, Javadpour J (2004). The effect of partially stabilized zirconia on the mechanical properties of the hydroxyapatite–polyethylene composites. Journal of Materials Science: Materials in Medicine.

[30] Abadi MBH (2010). Synthesis of nano β-TCP and the effects on the mechanical and biological properties of β-TCP/HDPE/UHMWPE nanocomposites. Polymer Composites.

[31] Fu S-Y, Feng X-Q, Lauke B, Mai Y-W (2008). Effects of particle size, particle/matrix interface adhesion and particle loading on mechanical properties of particulate–polymer composites. Composites Part B: Engineering.

[32] Hojo H, Toyoshima W, Tamura M, Kawamura N (1974). Short‐and long‐term strength characteristics of particulate‐filled cast epoxy resin. Polymer Engineering & Science.

[33] Young RJ, Beaumont PWR (1977). Failure of brittle polymers by slow crack growth. Journal of Materials Science.

[34] Homaeigohar SS, Sadi AY, Javadpour J, Khavandi A (2006). The effect of reinforcement volume fraction and particle size on the mechanical properties of β-tricalcium phosphate–high density polyethylene composites. Journal of the European Ceramic Society.

[35] Sadi AY (2006). The effect of partially stabilized zirconia on the biological properties of HA/HDPE composites *in vitro*. Journal of Materials Science: Materials in Medicine.

[36] do Amaral M, Roos A, Asua JM, Creton C (2005). Assessing the effect of latex particle size and distribution on the rheological and adhesive properties of model waterborne acrylic pressure-sensitive adhesives films. Journal of Colloid and Interface Science.

[37] Herrera‐Kao W, Aguilar‐Vega M (2009). Mechanical properties of latex blends films from polystyrene particles with different sizes in a butyl acrylate‐co‐styrene copolymer matrix. Polymer Engineering & Science.

[38] Robertson CG, Lin CJ, Rackaitis M, Roland CM (2008). Influence of Particle Size and Polymer−Filler Coupling on Viscoelastic Glass Transition of Particle-Reinforced Polymers. Macromolecules.

[39] Fortunati E (2013). Binary PVA bio-nanocomposites containing cellulose nanocrystals extracted from different natural sources: Part I. Carbohydrate Polymers.

